# The effects of different exercise loads in plyometric resistance training on respiratory and hormonal levels in female volleyball players

**DOI:** 10.3389/fphys.2025.1589080

**Published:** 2025-07-30

**Authors:** Korhan Kavuran, Ercan Tizar, Diclehan Oral, Ramazan Erdoğan, Baha Engin Çelikel, Tülay Ceylan, Süreyya Yonca Sezer, Baykal Karataş

**Affiliations:** ^1^ Bitlis University, School of Physical Education and Sports, Bitlis, Türkiye; ^2^ Dicle University, School of Physical Education and Sports, Diyarbakır, Türkiye; ^3^ Firat University, School of Physical Education and Sports, Elazığ, Türkiye; ^4^ Graduate Education Institute, Ondokuz Mayıs University, Samsun, Türkiye; ^5^ Faculty of Sport Sciences, Munzur University, Tunceli, Türkiye; ^6^ Ağrı İbrahim Çeçen University Faculty of Sports Sciences, Ağrı, Türkiye

**Keywords:** sports, post-activation performance enhancement, jump performance, voleyball, strength training resistance

## Abstract

**Background:**

The present study set out to ascertain the effects of combined exercises, consisting of resistance training and plyometric exercises at varying degrees of intensity, on biochemical and respiratory parameters in female volleyball players.

**Methods:**

The research group consisted of 20 professional female volleyball players who participated in national and international volleyball competitions. Participants were randomly divided into two groups: a control group (n = 10) that performed low-intensity exercises at 30%–50% intensity, and an experimental group (n = 10) that followed a high-intensity exercise programme at 60%–80% intensity. Blood samples and spirometric respiratory function values were collected before and after the 8-week exercise program. The biochemical analyses included the assessment of luteinizing hormone (LH), growth hormone (GH), insulin-like growth factor-1 (IGF-1), and total iron binding capacity. Respiratory function was analysed using forced vital capacity (FVC), forced expiratory volume in one second (FEV1), and forced expiratory flow at mid-expiration (FEF). The data were analysed using the SPSS statistical package.

**Results:**

The results demonstrated a decline in IGF-1, GH, and total iron-binding capacity levels, and an increase in LH levels, in the biochemical parameters of the control group. Additionally, FVC, FEV1, and FEF values exhibited an increase in the control group. Conversely, the experimental group demonstrated a significant increase in LH, GH, IGF-1, and total iron-binding capacity levels. With regard to respiratory parameters, an increase in FEF values was observed, whilst FVC and FEV1 values decreased. The present findings suggest that high-intensity plyometric resistance exercises have more pronounced effects on biochemical responses, but may trigger different adaptation mechanisms in respiratory capacit. The results of the study showed that there was a difference between the control group’s GH, IGF-1, FVC, and FEV1 data and the experimental group’s GH, IGF-1, FVC, and FEV1 data.

**Conclusions:**

In conclusion, plyometric resistance exercises at varying intensities influence both respiratory and biochemical parameters in female volleyball players. Accordingly, well-planned and individualised plyometric resistance training programmes are thought to enhance both the health and athletic performance of athletes.

## 1 Introduction

The effect of resistance training on improving biochemical and physiological parameters, particularly in female athletes, is supported by a series of studies. This type of training can have various effects on hormonal regulation and respiratory function, playing a crucial role in enhancing athletes’ performance (Hunter and Marshal, 2000; Gabriel et al., 2006; Sheel and Romer, 2012). The effect of strength training on hormones plays a fundamental role in muscle growth, strength development and recovery processes. During training, various hormones are released that stimulate muscle building and help reduce fat deposits. These hormones include growth hormone (GH), insulin-like growth factor-1 (IGF-1) and luteinizing hormone (LH) (Kraemer and Ratamess, 2005; Pullinen et al., 2002). Similarly, respiratory function determines the oxygen consumption capacity and efficiency of athletes during exercise. In sports such as volleyball, which require speed and endurance, a high oxygen transport capacity is necessary, making respiratory function a crucial component of athletic performance. Strength training enhances respiratory function by improving the efficiency of the respiratory system, and optimizing oxygen utilization. This, in turn, increases endurance and improves performance. This increases endurance and improves performance (Sheel and Romer, 2012; Inbar et al., 2000). In female volleyball players, training aimed at increasing both strength and endurance can lead to significant improvements in performance parameters. Specifically, high-intensity plyometric resistance training is effective in enhancing athletic performance in explosive strength-requiring sports like volleyball (Ramírez-Campillo et al., 2016). These types of training increase muscle strength and explosive movement ability, improving players’ jump height, speed and agility. In female volleyball players, strong respiratory function helps them endure higher intensity and longer durations during exercise, while optimizing hormonal responses also enhances muscle mass, strength, and endurance. Additionally, it is emphasized that low and high-intensity training can have different effects, making it important for training to be customized according to individual characteristics (Markovic and Mikulic, 2010).

Resistance training (RT) has been defined as a safe form of exercise for both athletes and non-athletes (Granacher et al., 2016). RT encompasses specialized physical conditioning methods that employ various resistance loads, different movement velocities and a variety of training modalities, including weight machines, free weights (barbells and dumbbells), elastic bands, Pilates balls and plyometric exercises (Faigenbaum and Myer, 2010).

Plyometric training can enhance athletes’ jumping performance skills as well as their biochemical and physical fitness parameters. Plyometrics is a form of exercise that involves repeated rapid stretching and contraction movements of muscles to increase power, commonly referred to as “explosive-reactive” resistance training (Ebben and Watts, 1998; Koźlenia and Domaradzki, 2024). Plyometric exercises are training techniques utilized by athletes to enhance power and explosiveness across various sports disciplines (Carvalho et al., 2014; Koźlenia and Domaradzki, 2023). Plyometrics consists of a rapid stretch (eccentric movement) of a muscle immediately followed by a concentric or shortening movement of the same muscle and connective tissues (Baechle and Earle, 2008). Plyometric training is based on two primary models that explain its underlying mechanisms. The first is the Mechanical Model, in which elastic energy is generated and stored in the muscles and tendons during a rapid stretch (Bosco et al., 1982). This stored energy is subsequently released when a concentric muscle action follows immediately after the stretch. The effect is similar to stretching a spring, which then recoils to return to its natural length. In this model, the muscles and tendons function as a series elastic component.

The second model is the Neurophysiological Model, which suggests that when a rapid stretch is detected in the muscles, an involuntary and protective response occurs to prevent excessive strain and potential injury. This response is known as the stretch reflex, which enhances the activity of the muscles subjected to stretching or eccentric contraction, thereby allowing them to generate more forceful movements. The result is a powerful braking effect and an increased potential for strong concentric muscle action. However, if the concentric contraction does not occur immediately after the prestretch, the potential energy produced by the stretch reflex is lost (i.e., if there is a delay between the downward bending and the subsequent jump, the counter-movement effect is diminished). Both the mechanical model (series elastic component) and the neurophysiological model (stretch reflex) are believed to contribute to the increased rate of force production during plyometric exercises (Gehri et al., 1998; Hunter and Marshal, 2000). Plyometric training is particularly relevant for athletes seeking to enhance their jumping ability and overall sports performance (Bal et al., 2012).

Regularly performed plyometric resistance training leads to increases in both muscular strength and muscle fiber cross-sectional area; however, these adaptations may develop over time (Balagopal et al., 2001; Hasten et al., 2000). Nevertheless, it has been suggested that the increase in strength cannot be attributed solely to muscle hypertrophy and that other mechanisms, such as neural adaptations, respiratory effort and biochemical factors, may also play a role (Gabriel et al., 2006; Gaurav and Singh, 2013; Singh, 2015; Chaabene et al., 2021). An efficient respiratory system is essential for athletes to meet the increased energy demands imposed by rhythmic muscular efforts during exercise (Singh, 2015). High respiratory function efficiency allows athletes to sustain higher training intensities and workloads (Inbar et al., 2000). In this context, improvements in respiratory function are particularly critical for optimizing elite performance in volleyball, a sport that requires endurance-based physical capacity. Enhancements in respiratory capacity are influenced by factors such as cardiac and skeletal muscle adaptations, the workload demands of specific playing positions and the intensity of training programs and exercises (Sheel and Romer, 2012).

Clinical research indicates that the positive effects of regular physical exercise are strongly influenced by workload intensity. While low-intensity workloads may be insufficient to elicit significant ergogenic adaptations, excessively high workloads that surpass an individual’s physiological threshold can impair recovery, potentially leading to decreased physical performance—a condition known as overtraining. Although numerous studies have explored training programs with varying loading intensities, research specifically investigating the effects of different intensities of plyometric resistance training in female volleyball players remains limited. Plyometric training is an effective exercise method primarily used to enhance performance parameters such as explosive strength, speed, agility and vertical jump. In sports like volleyball, which require frequent jumping and sudden changes of direction, this type of training plays a critical role in improving athletic performance. Particularly in female volleyball players, plyometric resistance training applied at varying intensities contributes to improvements in lower limb muscle strength, jump height, balance, coordination and the structure of muscle-tendon units. Depending on the intensity level, factors such as muscle damage, recovery time and adaptation responses may vary; therefore, it is important that training programs are tailored to individual characteristics. The literature suggests that while high-intensity plyometric training may lead to rapid performance gains, moderate- or low-intensity protocols tend to offer more sustainable long-term improvements (Markovic and Mikulic, 2010; Ramírez-Campillo et al., 2016). Therefore, this study aims to determine the effects of plyometric resistance training with different loading intensities on the biochemical and respiratory parameters of female volleyball players.

## 2 Materials and methods

### 2.1 Participants

The present study comprised 20 female athletes, with a mean age of 23.41 years (±1.23 years), a mean height of 183 cm (±1.44 cm), a mean body weight of 72.12 kg (±8.23 kg), a mean body BMI of 21.55 kg/m^2^ (±1.52 kg/m^2^) and a mean volleyball experience of 5.21 years (±1.4 years). The athletes trained a minimum of three times per week and participated in both national and international competitions. Participants in the study consisted of professional volleyball players with a defined sports background. Inclusion criteria required that all participants be free from any existing medical conditions or injuries, not under any form of medication, and demonstrate right-hand dominance (i.e., not ambidextrous). These criteria were established to ensure homogeneity within the sample and to eliminate potential variables that could influence physical performance outcomes. Power analysis was performed with G. Power 3.1 (G*Power software, Düsseldorf, Germany) to determine the number of participants required. The d value was 1.39 (α = 0.05, 1- β = 0.95) and the sample size was calculated as 18. The study was designed according to the principles of the Declaration of Helsinki (World Medical Association, 2013). Voluntary consent forms were obtained from subjects, and information about the study was provided. To circumvent potential issues, 25% more subjects were enrolled in each group. The study was conceptualised as a randomised, controlled experimental study, wherein participants were randomly assigned to two distinct groups: the experimental group and the control group. The randomisation of subjects into the two groups was determined by allocating them numbers between 1 and 20 through a computerised programme (https://www.randomizer.org/). The control group (n:10) performed a 30%–50% low-intensity plyometric resistance exercise programme, while the experimental group (n:10) performed a 60%–80% high-intensity plyometric resistance exercise programme. The exercise intensity for each participant in the running group was set at 50% of their heart rate (HR), calculated using the Karvonen formula:

$$\text{Target HR}=\left(\left[220-\text{age}-\text{resting HR}\right]\times \text{intensity}\right)+\text{resting HR}$$

HR was monitored from the first week of training using a telemetric heart rate monitor (Polar M400, Finland). The training protocol employed external load intensity, referring to the objective physical load applied to the athletes during the exercises. The exercise programme was characterised by a 20-s rest interval between movements and pulse rate was measured for each athlete using a Polar watch. The rationale for including only female volleyball players in this study is based on both methodological and physiological factors. It is known that gender-related differences in terms of exercise responses, hormonal profiles, musculoskeletal structure and physiological characteristics are well defined in the literature. Including both male and female athletes in the study may increase variability in the sample, make it difficult to interpret the results and reduce internal validity. Therefore, a homogeneous sample consisting of only female volleyball players was selected to increase the reliability and interpretability of the data obtained. In addition, volleyball is a sport widely practiced among female athletes. Therefore, examining exercise responses specifically for female athletes may provide valuable and targeted information in terms of training planning, performance development and reducing injury risks. Future studies may also include male athletes and allow for gender-based comparisons.

The subjects were invited to attend the sports science laboratory on three occasions. During the initial visit, they were informed about the study, and informed consent forms were obtained. The training programmes and performance tests were then administered. On their second visit, the subjects participated in the pre-workout performance tests. On their last visit, performance tests were taken 24–48 h after the last training session and recorded.

Prior to the commencement of the study, all participants were provided with a comprehensive information sheet and written consent was obtained in accordance with the ethical principles outlined in the Declaration of Helsinki. The exercise programme was devised and implemented with the consultation of expert coaches. The study was approved by Dicle University Social and Human Sciences Ethics Committee at its meeting dated 03.01.2023 and numbered 422269 (decision number E-14679147-663.05-423405).

### 2.2 Experimental design

The female volleyball players who participated in the study were required to visit the laboratory environment three times. During the initial visit, the experimental procedures were introduced and tested. Each subject was provided with a detailed explanation of the IMT (Inspiratory Muscle Training) procedure. On the subsequent visit, which occurred 1 week later, pre-workout measurements were taken, and the values were recorded. At the conclusion of the 6-week training program, the final measurements were taken during the third and final visits.

**FIGURE 1:**
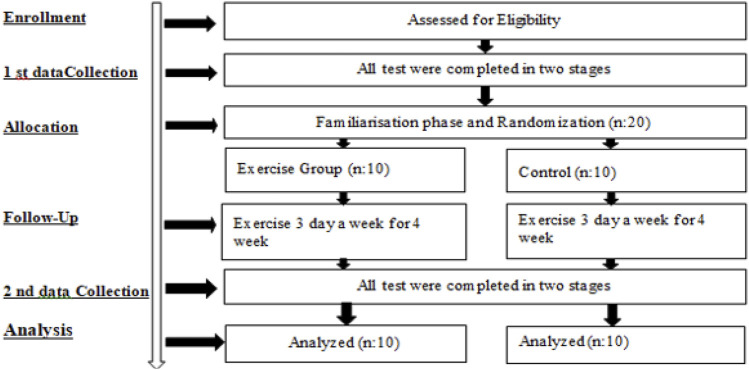
Experimental design.

In this study, volunteers were first assessed for eligibility, and those who met the inclusion criteria were enrolled in the study (Enrollment). All measurements were conducted in two stages on eligible participants (1st Data Collection). Subsequently, a familiarization phase was completed, and 20 participants were randomly assigned into two equal groups (Allocation): the Exercise Group (n = 10) and the Control Group (n = 10). Both groups participated in an exercise program three times per week for 4 weeks during the follow-up period (Follow-Up). At the end of the intervention, the same assessments were repeated for all participants and conducted in two stages once again (2nd Data Collection). In the data analysis phase, all participants in both groups (n = 10) were included in the analysis; therefore, no participant dropouts occurred during the study (Analysis) Figure 1.

### 2.3 Anthropometric measurements

The participants’ height was measured using a standardised height gauge (Seca, Germany) with an accuracy of 0.1 cm, while leaning against a wall without shoes. Their body weights were recorded before and after the 8-week sports training programme using a digital scale (Beurer, model GS27) with an accuracy of 0.1 kg. The participants wore minimal clothing and no footwear during the weighing process.

### 2.4 Vertical jump

In order to ascertain the potential impact of plyometric resistance training, a combination of applied resistance training and plyometric exercises, the countermovement jump (CMJ) heights were measured utilising a jumping mat (McCann and Flanagan, 2010). The CMJ was executed from a standing position, with feet positioned centrally on the jump mat and hands akimbo on the hips. Following an auditory signal, participants were instructed to flex their knees prior to jumping vertically to their maximum height, and to land in the centre of the jump mat. Video recordings of the subjects in the sagittal plane were used to verify that the knee flexion achieved the correct joint angle. Three trials were conducted, with a 60-s rest period between each trial. The highest value was then selected for data analysis (de Oliveira et al., 2019; Villalon-Gasch et al., 2022).

### 2.5 Pulmonary function tests

Forced Expiratory Flow at 25% of FVC FEF25, Forced Expiratory Volume in 1 Second FEV1, and Forced Vital Capacity FVC capacities were analyzed with an MGF Diagnostics CPFS/D USB (Saint Paul, Minnesota, United States) spirometer. Individuals with an Forced Expiratory Volume in 1 Second to Forced Vital Capacity Ratio FEV1/FVC value <75%, any chronic or pulmonary disease, medications that may affect lung function or a history of upper respiratory tract infection were excluded from the study. Pulmonary function measurements were performed while the patients were standing. During the tests, the subjects wore a nose clip to prevent air from escaping and were instructed to keep their lips tightly around the mouthpiece piece (American ThoracicSociety, 2002).

### 2.6 Biochemical analyses

Prior to the initiation of the exercise program, blood samples were collected from participants in the research group in a hospital setting by healthcare professionals in the morning after an overnight fast, for the purpose of biochemical analysis. Pre-cooled, EDTA-free yellow-capped (13 × 100 mm, 5 mL BD Vacutainer plastic tubes with SST gel) blood collection tubes were used for this procedure. Immediately following the completion of the 6-week exercise program, a second blood sample was obtained by healthcare staff present at the training facility using the same type of pre-cooled blood collection tubes. The samples were stored in special transport containers maintained at −25°C and delivered to the hospital laboratory for analysis. In the laboratory, the blood samples were separated into serum and plasma, and complete blood count analysis was performed using an Abbott Cell-DYN 3700R automatic hematology analyzer.

Blood samples were collected from all subjects between 8:00 and 10:00 in the morning following an overnight fast. Blood samples were obtained on two occasions, prior to and at the conclusion of the plyometric resistance training programme. Each subject underwent blood tests to measure luteinising hormone (LH), growth hormone (GH), insulin-like growth factor (IGF1) and total iron binding capacity (TIBC) serum levels. The blood samples from the athletes were obtained and analysed in a private hospital laboratory using a fully automated haemogram called “Coulter Stks” by specialists in a sitting position and at rest.

### 2.7 Exercise programme

**TABLE 1:** The training program applied to the volleyball players.

Exercises	Start-2 weeks	3–5 weeks	6–8 weeks
10 s plyometric air squat	×3	×3	×3
10 s plyometric jumping jack	×3	×3	×3
20 s two-legged plyometric box jump (20 reps, 50 cm)	×3	×3	×3
20 s plyometric squat	×3	×3	×3
20 s plyometric lunge	×3	×3	×3
20 s two-legged plyometric jumps with single-leg hops (60 cm)	×3	×3	×3
20 s leg extension		3 × 10	3×10
20 s hamstring curl		3 × 10	3 × 10
20 s calf raises		3 × 10	3 × 10
20 s deadlift		3 × 10	3 × 10
20 s plank	3 × 10	3 × 10	3 × 10

### 2.8 Statistical analysis

Statistical analyses were performed via SPSS (Version 21.0 for Windows, Chicago, IL, United States) software, with the statistical significance set at 0.05. The Shapiro‒Wilk normality test was performed to determine the homogeneity of the sample. The pre-test and post-test differences of each group were determined via the paired comparison test (paired t-test), and the post-test and pre-test difference values were determined via one-way analysis of variance. In addition, in the comparison of paired groups, the effect size was calculated according to Hedges’ g (Hedges, 1981). Moreover, it was interpreted as follows: 0–0.19 insignificant, 0.20–0.59 small, 0.6–1.19 moderate, 1.20–1.99 large, and ≥2.00 very large.

## 3 Results

**FIGURE 2:**
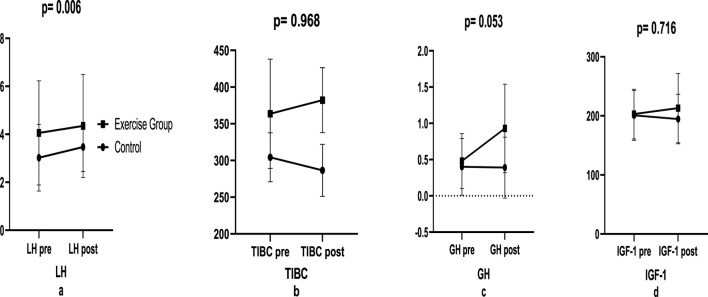
Biochemical changes in exercise pre-post.

In the biochemical parameters before and after plyometric resistance training, LH hormone increased in Control (14.9%; e.s.: 0.369) and Exercise group (7.14%; e.s.: 0.134) (p = 0.006, Figure 2a). In TIBC, there was a decrease in Control (−5.79%; e.s.:0.219) and an increase in Exercise group (5.23%; e.s.:0.302) (p = 0.986; Figure 2b). In GH, there was a decrease in Control (−2.5%; e.s.: 0.025) and an increase in Exercise group (95.74%; e.s.: 0.896) (p = 0.053, Figure 2c). In IGH-1, there was a decrease in Control (−3.09%; e.s.: 0.045) and an increase in Exercise group (4.93%; e.s.: 0.196) (p = 0.716, Figure 2d; Table 4).

**TABLE 2:** Biochemical comparison between groups.

Parameters		Control	e.s.	%	Exercise group	e.s.	%	p
		X ± S.D			X ± S.D			
LH	Pre	3.02 ± 1.39	0.369	14.9	4.06 ± 2.17	0.134	7.14	0.006
	Post	3.47 ± 1.02↑			4.35 ± 2.15↑			
GH	Pre	0.40 ± 0.38	0.025	−2.5	0.47 ± 0.38	0.896	95.74	0.053
	Post	0.39 ± 0.41			0.92 ± 0.60↑			
IGF-1	Pre	200.80 ± 42.29	0.045	−3.09	203.10 ± 42.10	0.196	4.93	0.716
	Post	194.60 ± 42.07↓			213.10 ± 58.61			
TIBC	Pre	304.20 ± 33.30	0.219	−5.79	363.60 ± 74.31	0.302	5.23	0.986
	Post	286.60 ± 35.48↓			382.10 ± 44.33			

p < 0.05, e.s.: effect size (Hedges’ g). ↑: Increased; ↓: Decreased.

**FIGURE 3:**
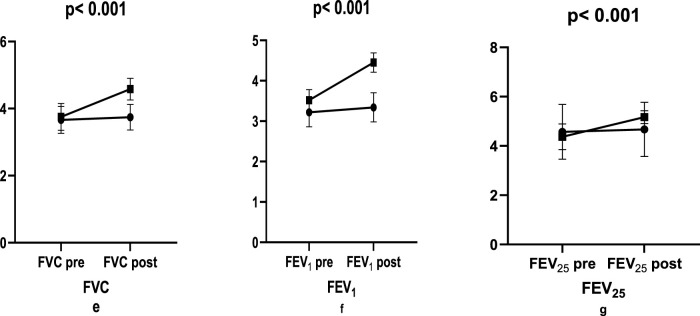
Respiratory changes in exercise pre-post.

In the study, there was an increase in FVC before and after plyometric resistance training in Control (2.19%; e.s.:0.210), Exercise group (21.87%; e.s.:2.298) (p < 0.001; Figure 3e). There was an increase in FEV1 in Control group (4.05%; e.s.:0.361) and Exercise group (26.14%; e.s.:3.301) (p < 0.001, Figure 3f). In FEF, there was an increase in Control (2.41%; e.s.:0.100) and Exercise group (18.35%; e.s.:1.946) (p < 0.001, Figure 3g; Table 3).

**TABLE 3:** Comparison of respiratory parameters between groups.

Parameters		Control	e.s.	%	Exercise group	e.s.	%	p
		X ± S.D			X ± S.D			
FVC	Pre	3.66 ± 0.39	0.210	2.19	3.5 ± 0.39	2.298	21.87	<0.001
	Post	3.74 ± 0.37↑			4.57 ± 0.32↑			
FEV_1_	Pre	3.21 ± 0.36	0.361	4.05	3.52 ± 0.25	3.301	26.14	<0.001
	Post	3.34 ± 0.36			4.44 ± 0.23↑			
FEF_25_	Pre	4.56 ± 1.10	0.100	2.41	4.36 ± 0.52	1.946	18.35	<0.001
	Post	4.67 ± 1.09↑			5.16 ± 0.26↑			

p < 0.05, e.s.: Effect size (Hedges’ g). ↑: Increased; ↓: Decreased.

**FIGURE 4:**
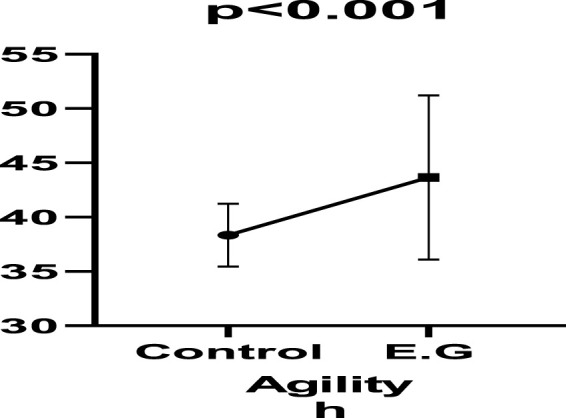
Agility in exercise pre-post.

In the study, there was an increase in vertical jump performance before and after plyometric resistance training in Control (11.29%; e.s.:1.430), Exercise group (28.98%; e.s.:2.829) (p < 0.001; Figure 4h). The study concluded that the incorporation of high-intensity plyometric resistance training in the exercise group, in addition to the training programmes, resulted in a 17.69% improvement in vertical jump performance when compared to low-intensity plyometric resistance training in the control group among female volleyball players (p < 0.001) Table 4.

**TABLE 4:** Comparison of vertical jump between groups.

Parameters		Control	e.s.	%	Exercise group	e.s.	%	p
		X ± S.D			X ± S.D			
Vertical jump	Pre	36.30 ± 2.91	1.430	11.29	38.30 ± 2.98	2.829	28.98	<0.001
	Post	40.40 ± 2.76↑			49 ± 4.85↑			

↑: Increased; ↓: Decreased.

## 4 Discussion

The adaptation of high-intensity training (HIT) is of significant importance, as is the creation of a number of metabolic changes in the organism. However, the effect of different loading intensities on the biochemical and respiratory parameters of the organism is not yet fully understood. Therefore, this study was conducted to determine the effect of plyometric resistance training at different loading intensities on the biochemical and respiratory parameters of athletes.

The findings of the study indicate a significant decrease in IGF-1 levels and an increase in LH levels in the control group that underwent low-intensity plyometric resistance training. Additionally, a decrease in GH and total iron-binding capacity (TIBC) levels was observed. Regarding respiratory parameters, a significant increase in FVC and FEF values was observed, while the increase in FEV1 values was not significant. In the high-intensity plyometric resistance training group, a significant increase in LH and GH levels was observed, while non-significant increases were detected in IGF-1 and TIBC levels. These results suggest that high-intensity training had a more pronounced effect on hormone levels, but limited effects on IGF-1 and TIBC parameters.

In our study, a significant increase in FEF values was observed, while significant decreases were noted in FVC and FEV1 values. This suggests that the effects of low-intensity training plyometric resistance training on the respiratory system may be complex and multifaceted. The increase in FEF values indicates an improvement in airway patency and the function of the smaller airways, whereas the decreases in FVC and FEV1 values may be attributed to individual differences, training intensity, or the duration of the program. However, these results contrast with the findings of Gökhan et al. (2011), who reported significant increases in FEV1, FVC, VC, and MVV values following swimming exercises. This discrepancy may be due to the type of exercise employed. Swimming involves the synchronized use of the entire body’s muscles and enhances breath control, whereas plyometric resistance training primarily targets the musculoskeletal system and does not directly engage the respiratory muscles. Therefore, swimming may have a more pronounced positive effect on respiratory function. Additionally, the study by Souissi et al. (2020) found that 15/15 interval exercises caused less oxidative stress in the body compared to 30/30 interval exercises. This finding aligns with our study, in which high-intensity training produced more noticeable changes in hormonal and biochemical parameters. In particular, the increase in hormones such as LH and GH following high-intensity exercise may indicate higher physiological stress and adaptation responses in the body.

In our study, it was observed that high-intensity plyometric resistance training led to more pronounced changes in hormonal and biochemical parameters. This finding is consistent with the study conducted by Prasertsri and Boonla (2021), who also reported that although high-intensity interval training enhanced physical performance, it did not have as favorable an effect on oxidative stress as resistance training. Similarly, in our study, high-intensity training resulted in an increase in GH and LH levels, while no significant changes were observed in parameters such as IGF-1 and TIBC. This highlights the complexity of oxidative stress and adaptation processes. The study by Zerin et al. (2015) reported an increase in MVV following a specific exercise program, but no significant changes in basic respiratory parameters such as FVC and FEV1. This finding aligns with our study, in which we detected a significant increase in FEF values, but not in FVC and FEV1. This suggests that factors such as the type and duration of training, as well as participant characteristics, play a critical role in the effects on respiratory functions. Additionally, the findings of Sukatan et al. (2022), who emphasized that respiratory muscle warm-ups significantly improve respiratory parameters, are noteworthy. In our study, no specific respiratory muscle warm-up protocol was applied, which may explain the decrease observed in FVC and FEV1. Therefore, future studies may benefit from including respiratory muscle activation before training to positively influence these parameters.

Various studies in this field have revealed the effects of exercise type, duration, and intensity on athletes’ physiological parameters from different perspectives. İnce et al. (2022) reported in their study conducted among students at the Faculty of Sport Sciences that spirometric measurement values significantly differed depending on gender and performance levels. This finding indicates the influence of individual differences on respiratory capacity. Similarly, Usman and Shenoy (2015) demonstrated that the exercise program they implemented had a positive effect on both the respiratory functions and vertical jump performance of athletes. This supports the idea that regular and structured exercise can enhance respiratory capacity, thereby contributing to physical performance. However, it is not possible to claim that every exercise program yields positive effects. Indeed, the study by Škrgat et al. (2018) revealed that high-intensity training induced oxidative stress in athletes’ respiratory functions. This suggests that intense training, especially when poorly managed or lacking adequate rest periods, can have adverse effects on the body. Therefore, it can be concluded that training intensity must be carefully planned. On the other hand, Kahraman et al. (2023) found that acute repetitive sprint training affected certain biochemical parameters (such as glucose and creatine levels), but did not result in significant changes in parameters like urea, albumin, AST, ALT, LDH, and GGT. These findings suggest that short-term, high-intensity training can influence some markers of energy and muscle metabolism, while not necessarily causing systemic effects such as those related to liver function or protein metabolism. Overall, when these studies are evaluated collectively, it can be concluded that the effects of exercise on respiratory functions and biochemical parameters vary depending on the type, duration, intensity of the exercise, as well as individual characteristics. Therefore, in aiming to enhance athletic performance, training programs should be planned with consideration of individual traits and physiological responses.

The physiological effects of exercise on athletes have been examined in a multidimensional manner through various studies. Particularly, the biochemical and hormonal changes that occur in the body depending on the intensity, duration, and type of exercise have been reported with differing results in the literature. Araneda et al. (2014) observed an increase in oxygen- and nitrogen-derived pro-oxidative parameters following a 50-min high-intensity exercise in physically active individuals. This finding suggests that high-intensity training may induce oxidative stress, indicating that antioxidant defense mechanisms play a significant role in this process. These results align with those of Škrgat et al. (2018), who also found that intense training induced oxidative stress in the respiratory functions of athletes. On the other hand, studies focusing on hormone levels have revealed noteworthy outcomes. Roli et al. (2018) reported that seasonal variations in hormone levels such as testosterone, cortisol, growth hormone, and IGF-1 in elite volleyball players were above the reference values. This indicates that high-level training loads may influence hormonal balance, emphasizing the importance of individualized monitoring. However, in the study conducted by Portal et al. (2011), although the exercise program led to improvements in body composition, muscle strength, and both aerobic and anaerobic capacity, it did not result in significant changes in anabolic hormones (GH, IGF-I, testosterone), catabolic hormones (cortisol), or inflammatory markers (IL-6 and IL-1ra). These conflicting results suggest that the effects of exercise on hormonal responses may depend not only on exercise parameters but also on individual factors such as training history, age, gender, and nutritional status. When all these studies are evaluated collectively, it becomes clear that the effects of exercise on the body are highly complex and multifaceted. While some types of exercise may enhance respiratory and performance capacity, others may lead to oxidative stress or hormonal fluctuations. Therefore, training programs for athletes should be designed with careful consideration of individual differences, performance goals, and health conditions.

The effects of exercise on hormonal responses and metabolic markers have been examined from various perspectives in numerous studies within the field of sports sciences. In particular, the type, duration, intensity of the exercise, and whether it is combined with supportive applications can directly influence the resulting physiological outcomes. Mejri et al. (2005) reported that submaximal exercise influenced the levels of growth hormone (GH) in athletes, but did not lead to a significant change in insulin-like growth factor-1 (IGF-1) levels. This finding highlights the sensitivity of hormonal responses to exercise intensity and indicates that not all anabolic markers respond similarly. Similarly, Portal et al. (2011) also observed that exercise had no significant effect on anabolic hormones such as GH and IGF-1. These results suggest that hormonal responses are closely related not only to individual differences but also to specific exercise parameters. On the other hand, the study by Jaworska et al. (2018) demonstrated that a volleyball training program supported by whole-body cryostimulation had positive effects on both metabolic and neurological parameters. The reduction in glucose levels indicates the contribution of exercise to energy utilization, while the increase in IGF-1 and brain-derived neurotrophic factor (BDNF) levels suggests that such supportive methods may offer potential benefits in areas such as neuroplasticity and muscle development. This finding also aligns with the results of Kahraman et al. (2023), who reported that short-term sprint training had an effect on certain metabolic parameters (e.g., glucose levels).

This study suggests that high-intensity plyometric resistance training can significantly improve the vertical jump performance of female volleyball players. The results are noteworthy when compared to similar studies in the literature. Firstly, the effects of plyometric training on vertical jump have been confirmed in many other studies. For example, a study by Şahin et al. (2025) reported that plyometric training led to a significant improvement in the jump performance of volleyball players. Similarly, Beato et al. (2018) found that plyometric training was effective in increasing vertical jump height in both male and female athletes. These studies support the effectiveness of high-intensity plyometric resistance training in enhancing athletes’ explosive power. Additionally, another study observed that plyometric training provided more pronounced benefits in increasing jump height compared to low-intensity training (McBride et al., 2002). McBride et al. (2002) found that high-intensity plyometric training resulted in larger jump improvements in a shorter period of time. This finding suggests that higher intensity provides greater stimuli and more training volume, leading to performance gains. However, some studies have reported that low-intensity plyometric training can also have positive effects on jump performance. However, it has been emphasized that these effects are generally less pronounced and require longer-duration programs compared to high-intensity training (León Muñoz et al., 2015). In this study, the participants’ dietary habits and supplement use were not systematically controlled. This situation is considered as a limitation that may have a potential effect on biochemical parameters. In particular, parameters such as growth hormone (GH), IGF-1 and total iron binding capacity may be affected by individuals’ daily macro and micronutrient intake and/or supplement use. However, at the beginning of the study, participants were informed that they should not use any medication or ergogenic supplement (vitamin, mineral, protein powder, creatine, etc.) and were asked to declare that they did not use such products within the scope of voluntary consent forms. However, these statements were not confirmed by objective biochemical analyses. Therefore, it should be considered that the products that the participants may have used consciously or unknowingly may have an effect on the study results. Future studies can control the effect of these variables more clearly by supporting them with nutritional records, diet diary follow-up or data on supplement use from serum levels. The findings obtained in this study should be interpreted in the context of this limitation and caution should be exercised, especially in attributing changes in hormonal parameters solely to exercise.

The present study is subject to certain limitations. Firstly, the dietary habits of the female volleyball players were not controlled. Secondly, the study was limited to female volleyball players. Additionally, due to restricted access to elite athletes under competition conditions, the sample size was relatively small. Further research with larger sample sizes is required to better understand the effects of plyometric resistance exercises on female volleyball players and related sports.

## 5 Conclusion

It was thus determined that varying loading intensities in the context of plyometric resistance training, as implemented on female volleyball players, exerted an influence on respiratory function, vertical jump performance, and biochemical parameters. In accordance with these findings, it is hypothesised that plyometric resistance training will exert a favourable impact on the health and sporting performance of athletes.

## Data Availability

The original contributions presented in the study are included in the article/supplementary material, further inquiries can be directed to the corresponding author.
